# Modified mini-open SRS-Schwab grade 4 osteotomy combined with percutaneous pedicle screws fixation in post-traumatic thoracolumbar kyphosis

**DOI:** 10.1186/s12891-020-03666-8

**Published:** 2020-09-28

**Authors:** Wubo Liu, Yiwei Zhao, Suomao Yuan, Yonghao Tian, Xinyu Liu

**Affiliations:** 1 Department of Orthopedics, Cheeloo College of Medicine, Qilu Hospital of Shandong University, Wenhua Road 107#, Jinan, Shandong Province 250012 People’s Republic of China; 2 Cheeloo College of Medicine, Shandong University, Jinan, Shandong 250012 People’s Republic of China

**Keywords:** Post-traumatic, Thoracolumbar kyphosis, Percutaneous pedicle screws, Schwab grade 4 osteotomy

## Abstract

**Background:**

We aimed to analyze the clinical results of Schwab grade 4 osteotomy combined with percutaneous pedicle screws (PPS) fixation for treatment of post-traumatic thoracolumbar kyphosis (PTK).

**Methods:**

Thirty four patients with PTK (group A) were included in our study. The average age was 54.9 ± 3.3 years. All patients had severe back pain with 8.6 ± 1.3 VAS scores. The affected level was T12 in 16 patients and L1 in 18 patients. The average preoperative regional kyphosis angle (RKA) was 30.7 ± 6.00. Three patients had neurological dysfunction with ASIA grade D. All patients underwent Schwab grade 4 osteotomy combined with PPS fixation. The control group (Group B) were 26 PTK patients treated with Schwab grade 4 osteotomy and open pedicle screws fixation in our institution.

**Results:**

Operation time in groups A and B was 280 ± 50 min and 210 ± 30 min, respectively (*P* < 0.05). Estimated blood loss in groups A and B was 310 ± 70 ml and 630 ± 40 ml, respectively (P < 0.05). No cerebral spinal fluid leakage, segmental nerve function damage, and other complications observed during and after the operations in both groups. RKA, SVA, and LL improved significantly after surgery in both groups (*P* < 0.05). The average correction rate in groups A and B was 64.5 and 66.3% (*P* > 0.05). CT showed that the misplacement rate in groups A and B was 5.5 and 6.6% (P > 0.05). The average follow-up in groups A and B was 25.2 ± 7.6 months and 30.6 ± 2.7 months. No fracture and other complications were observed in both groups. Solid bone fusion was showed in all cases at 6 months follow-up. In groups A and B, all patients with preoperative neurological dysfunction recovered to ASIA grade E at the last follow-up. The VAS score of back pain improved significantly from 8.6 ± 1.3 to 1.6 ± 1.0 at the last follow-up in group A (*P* < 0.05), while it improved significantly from 8.3 ± 1.2 to 3.0 ± 1.1 at the last follow-up in group B (P < 0.05). VAS of back pain was better in group A than that in group B.

**Conclusion:**

Schwab grade 4 osteotomy combined with percutaneous pedicle screws fixation is a minimally invasive, safe and effective method for PTK treatment.

## Background

Post-traumatic kyphosis (PTK) at thoracolumbar level is a common clinically spinal deformity, which is usually associated with severe specific mechanical neurological dysfunction or low-back pain. For some severe post-traumatic thoracolumbar kyphosis, the essential factor for a successful corrective surgery is to select appropriate osteotomies based on patients’ individual characteristics.

The main surgical methods for kyphosis correction include pedicle subtraction osteotomy (PSO), Smith-Petersen osteotomy (SPO), vertebral column resection (VCR), closing-opening wedge osteotomy (COWO), and vertebral column decancellation [1–10]. Schwab et al. [11] classified spinal osteotomy technique into 6 grades according to the osteotomy site: grade 1 is partial facet joint resection such as SPO osteotomy; grade 2 is complete facet joint resection such as Ponte osteotomy; grade 3 is pedicle and partial body resection such as PSO; grade 4 is pedicle, partial body, and disc resection; grade 5 is complete vertebra and disc resection such as VCR and grade 6 is multiple adjacent vertebrae and disc resection.

For post-traumatic kyphosis cases with severe bone compression, such as grade 3 in Genant spinal deformity index (severe fracture, with loss of height greater than 40%.) [12], Schwab grade 3 osteotomies are not suitable for correction of kyphosis. Comparing with grade 5 osteotomy, grade 4 osteotomy has a few advantages for post-traumatic thoracolumbar kyphosis, such as less surgical trauma, lower complication rates, and easier stability reconstruction [6–8]. Grade 4 osteotomy was first reported by Scudese et al. in 1963. Scudese et al. reported a modified SPO with additional removal of the superior portion of the affected vertebral body and superior disc [13]. Murrey used grade 4 osteotomy based on egg-shell technique to treat spine deformity, tumor, and infection cases, which acquired satisfactory clinical results [14]. The affected vertebral body’s shape is usually like a trapezoid or triangle (lower height at the anterior vertebral body), which makes it easier to remove the upper level intervertebral disc and cancellous bone of the vertebral body through the pedicle. Combined with egg-shell technique, the risk of neurological injury and blood loss during the grade 4 osteotomy procedure can be further reduced [10].

Percutaneous pedicle screw (PPS) has been developed for more than 15 years. PPS can significantly decrease in the operation time, intraoperative blood loss, blood transfusion, opioid doses, length of hospitalization, and postoperative visual analog scale (VAS) score. Until now, PPSs have been widely applied to treat thoracic and lumbar spine trauma, degenerative diseases, and scoliosis [15–18]. Minimally invasive surgery (MIS) techniques are becoming a more common treatment for correcting the deformity. Multilevel lateral transpsoas lumbar interbody fusion plus PPSs, or multilevel transforaminal lumbar interbody fusion plus PPSs was successfully used for adult scoliosis cases [17]. However, there are few clinical reports of PPS in kyphosis correction. In this paper, we reported the clinical efficacy of mini-open SRS-Schwab grade 4 osteotomy (egg-shell technique) in a combination of PPS for the treatment of post-traumatic thoracolumbar kyphosis.

## Methods

### Inclusive and exclusive criteria

Our research was approved by the ethics committee of Qilu Hospital of Shandong University. All participants agreed with the data and publication of the manuscript. The inclusive criteria were post-traumatic thoracolumbar kyphosis which: (1) the Cobb angle exceeding 30 in X-ray sagittal plane; (2) had severe back pain (Visual analog scale (VAS) ≥ 7) w/o neurological deficit; (3) was not suitable for PVP (Percutaneous Vertebroplasty)/PKP (Percutaneous kyphoplasty); (4) failed to respond for conservative treatment for more than 3 months.

The exclusion criteria were as follows: (1) the kyphosis caused by infection, tumor or any other diseases; (2) with severe osteoporosis (BMD t ≤ − 3.0 SD); (3) with severe cardiovascular, cerebrovascular or any other diseases, which could not tolerate surgery.

### Patient population

From January 2014 to January 2018, 50 cases of post-traumatic thoracolumbar kyphosis were included. According to the Genant spinal deformity index [11], all fractures are classified as wedge, grade 3. In group A, a total of 34 patients (13 males, 21 females) with PTK at the thoracolumbar level underwent mini-open SRS-Schwab grade 4 osteotomy (egg-shell technique) in combination with PPS for treatment of post-traumatic thoracolumbar kyphosis. The control group (Group B) were 26 patients treated with the Schwab grade 4 osteotomy and open pedicle screws fixation.

The average age, VAS score of back pain, affected levels, the average time from initial fracture to admission, and the American Spinal Injury Association (ASIA) Impairment Scale grade showed in Table 1.

**Table 1:** The demographic of group A and B

	Group A	Group B
Cases	34	26
Average age (years)	54.9 ± 3.3	55.6 ± 5.7
Gender		
males	13	10
females	21	16
VAS of back pain	8.6 ± 1.3	7.8 ± 1.4
Affected level		
T12	16	12
L1	18	14
Time from initial fracture to admission (months)	6.2 ± 4.3	5.7 ± 2.5
ASIA scale		
D	3	4
E	31	22

### Surgical procedure

**Fig. 1:**
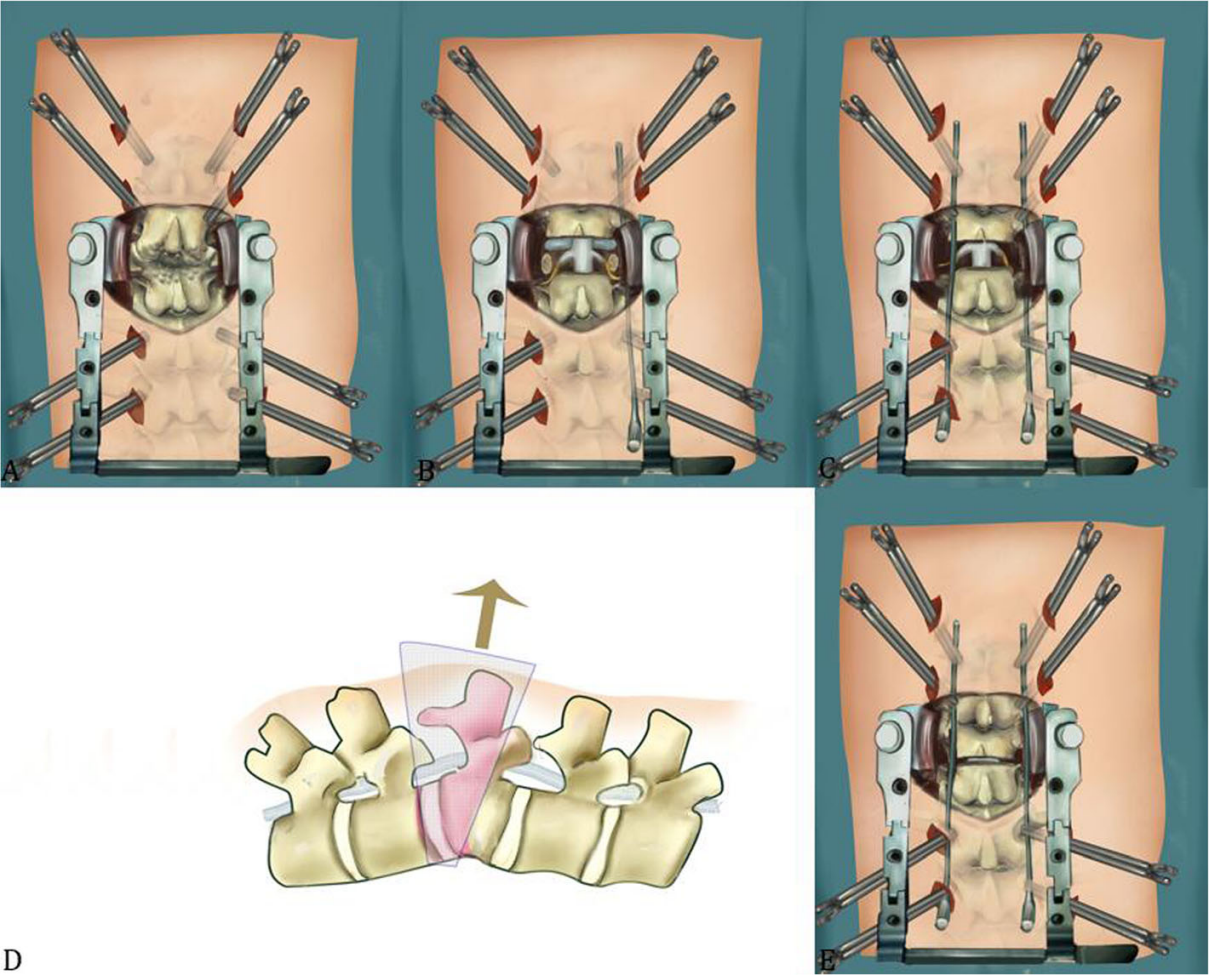
Schematic of Schwab grade 4 osteotomy and combined with percutaneous pedicle screws fixation in post-traumatic thoracolumbar kyphosis. **a**. A posterior midline incision was used to expose the lamina and spinous process of the osteotomy level. **b**. The lamina and spinous process were removed. **c**-**d**. By using the eggshell technique, the upper part of vertebral body, the disc and endplate above the osteotomy level were removed. **e**. The pre-curved rods with appropriate length, were placed percutaneously and kyphosis was corrected by closing the osteotomy site

**Fig. 2:**
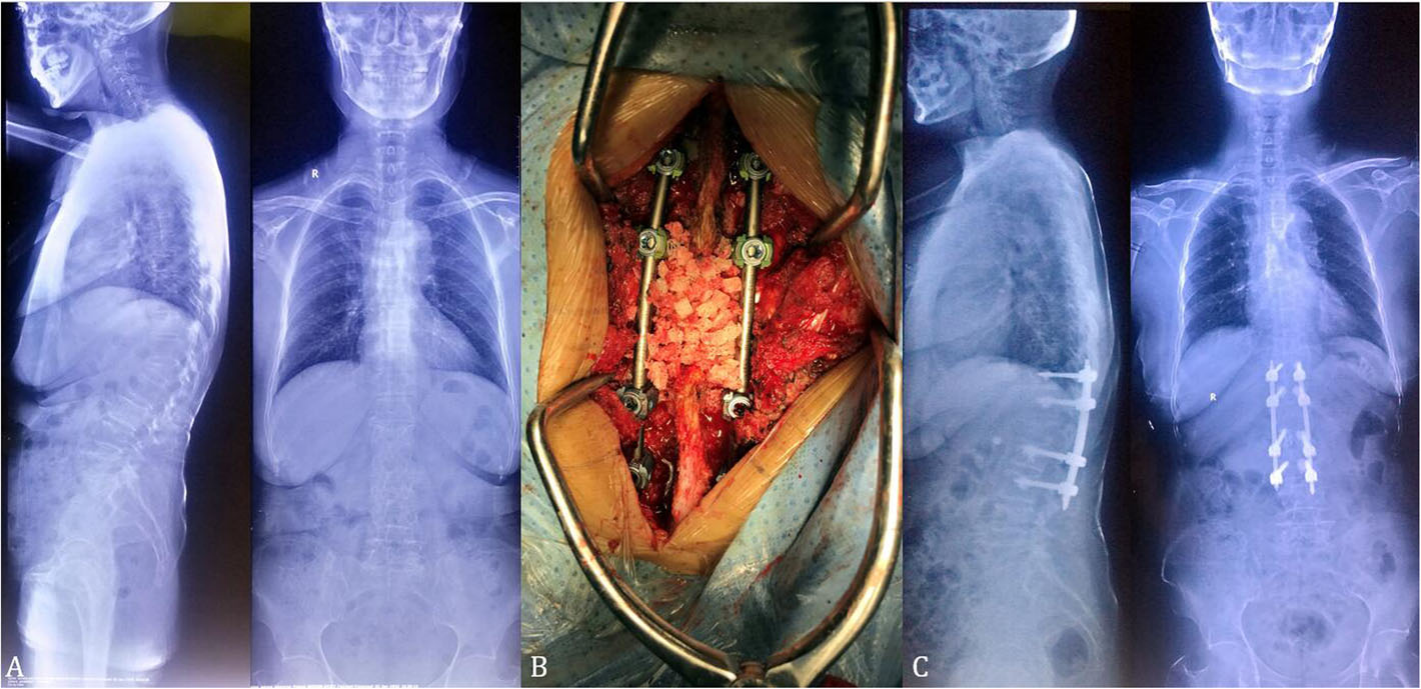
A 69-year-old female with severe back pain (VAS score of 7) due to L1 post-traumatic kyphosis. **a**. The X-rays showed L1 old compression fracture, RKA was 32.7^0^. **b**. the patient underwent Schwab grade 4 osteotomy and internal fixation in traditional method. **c**. Postoperative X-ray (2-year follow-up) showed satisfactory correction with RKA of 12^0^

The surgical procedure was briefly described below. First, the patients were in a prone position after general anesthesia. In group A, a Jamshidi needle was inserted into the pedicles of the upper and lower two segments under the fluoroscopic guidance. Then a K-wire was passed into the pedicles through the Jamshidi trocar, and a cannulated polyaxial screw was advanced over the K-wire. Routine polyaxial PPSs (33 cases) or MIS bone cement reinforced pedicle screw system (1 case) with soft extended slice (Zina MIS Spine System, Shanghai Sanyou Medical CO., LTD) were used in this series. Then, a posterior midline incision (5-8 cm) was made to expose the lamina and spinous process of the affected level. The grade 4 osteotomy with egg-shell technique was performed in a similar way as previously described [10]. Briefly, after removing the spinous process and lamina, the left pedicle was exposed and decancellated by a high-speed drill. Then, the pedicle cortical bone was removed to gain access to the vertebral body, protect the dura and the nerve roots. With this eggshell technique, a curette and a drill were used to decancellate the upper part of the vertebral body on the left side. Then the cortical bone of the vertebral body on the left side was burred away gradually. A temporary fixing rod was placed to maintain the stability of the spine. A similar procedure was performed on the right side. Then the disc and endplate above the osteotomy were removed from both sides. At last, to collapse the bone into the cavity, an L-shaped posterior wall impactor and a burr were used to remove the portion of the vertebral body underlying the anterior spinal canal. The contoured rods with appropriate length and sagittal curve were placed percutaneously and were fixed on the right side. Under the protection of the connecting rods on both sides, we alternately compressed the temporary rod and long connecting rod carefully, and gradually closed the osteotomy site until the posterior elements are touching (Fig. 1). After the osteotomy, autogenous bone grafts were widely spread on the exposed lamina for fusion (Fig. 2b). If necessary, titanium mesh or PEEK cage filled with autograft would be placed through the left or right side if there was a large bone defect at the osteotomy site after correction.

During the whole operation, intraoperative blood salvage and inter-operative Monitoring (IOM) including motor evoked potentials (MEPs) and somatosensory evoked potentials (SEPs) were used throughout the surgeries. Two days after operation, patients were encouraged to walk with the help of braces (Fig. 2).

**Fig. 3:**
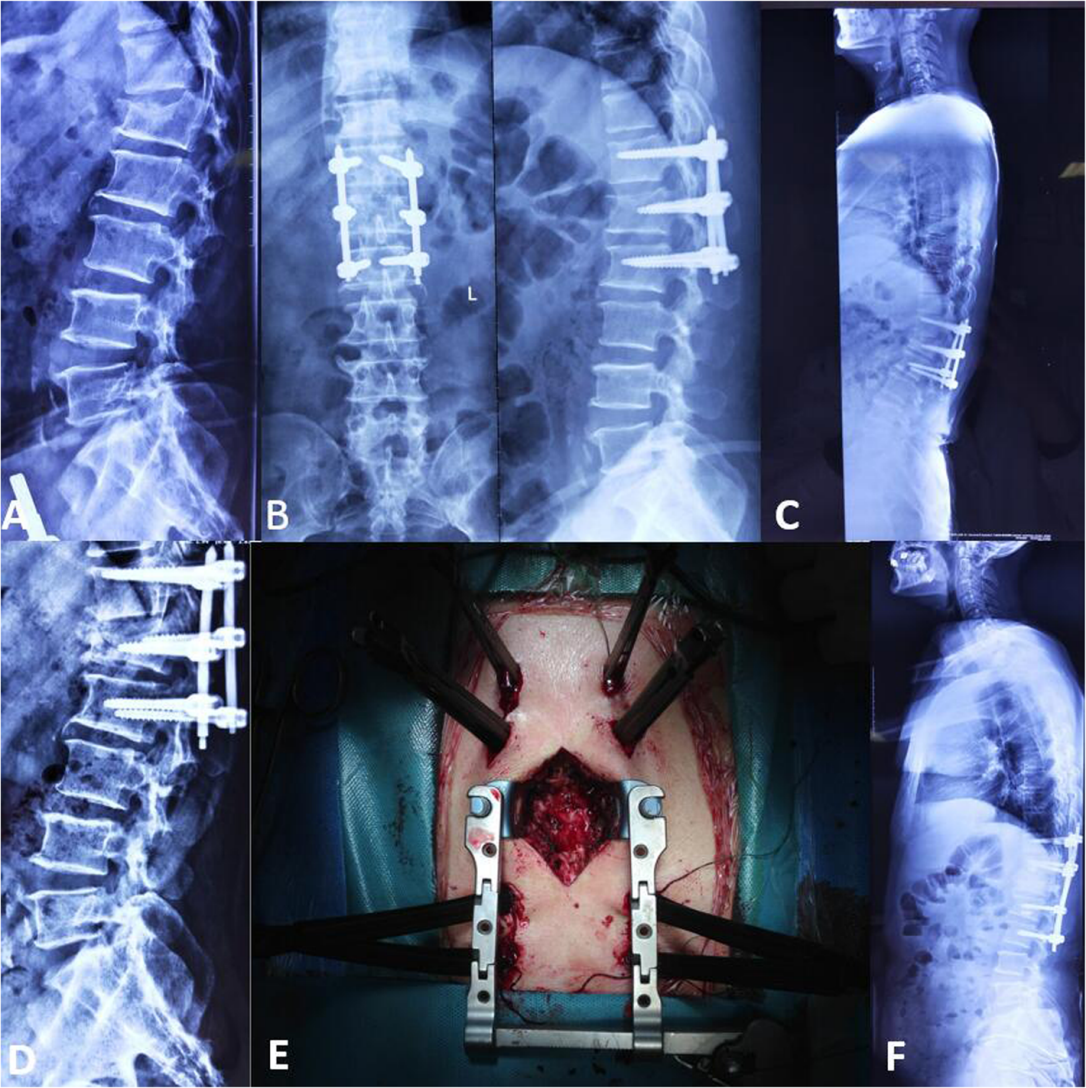
A 59-year-old male with severe back pain (VAS score of 9) due to L1 post-traumatic kyphosis. **a**. The X-rays showed L1 compression fracture. b. the patient underwent reduction and PPS internal fixation at local hospital. **c**-**d**. Nine months after the first surgery, the reduced fracture collapsed with severe back pain. The X-rays showed the RKA was 33.4^0^and loosening of the bilateral L_2_ PPSs. **e**. Intraoperative common PPSs with soft extender at T_11_ and L_3_, while cement PPSs with soft extender were used at T12 and L_2_ at the revision surgery. A 5 cm incision at median line was used for Schwab grade 4 osteotomy. **f**. Postoperative X-ray (1-year follow-up) showed satisfactory correction with RKA of 12.4^0^

For each patient in Group B, to expose the surgical levels, the paravertebral muscles were detached from the lamina and spinous processes through the midline posterior approach, and the pedicle screws were inserted into two or three adjacent upper and lower segments of the proposed osteotomy site. Then Schwab grade 4 osteotomy and deformity correction were performed similarly with group A (Fig. 3).

### Imaging, neurological function and pain evaluation

VAS score was used to assess pre- and post-operative back pain (0 indicating no pain and 10 representing the worst pain). ASIA grade was used to assess the pre- and post-operative neurological function.

**Fig. 4:**
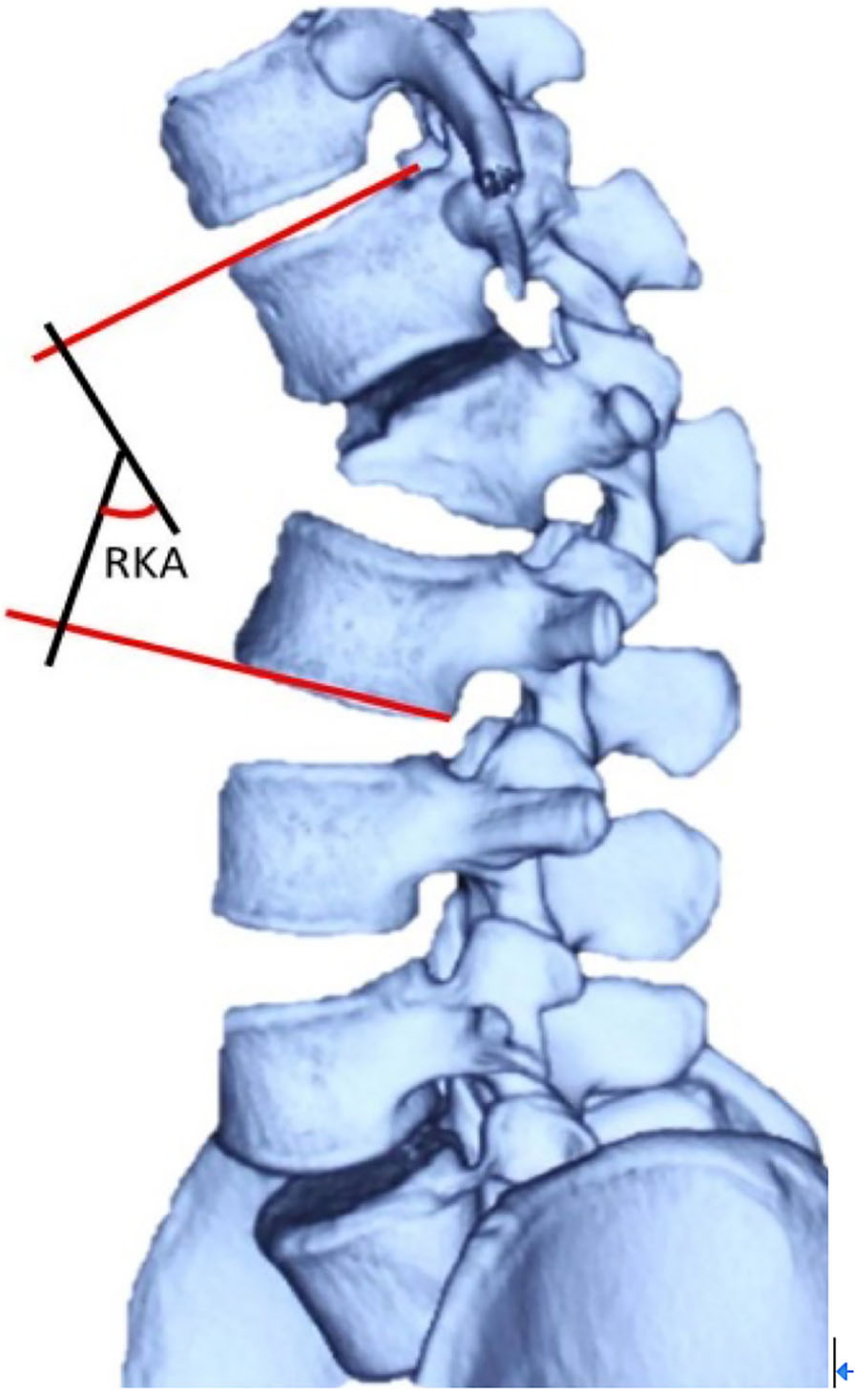
Measurement of regional kyphosis angle (RKA)

**Fig. 5:**
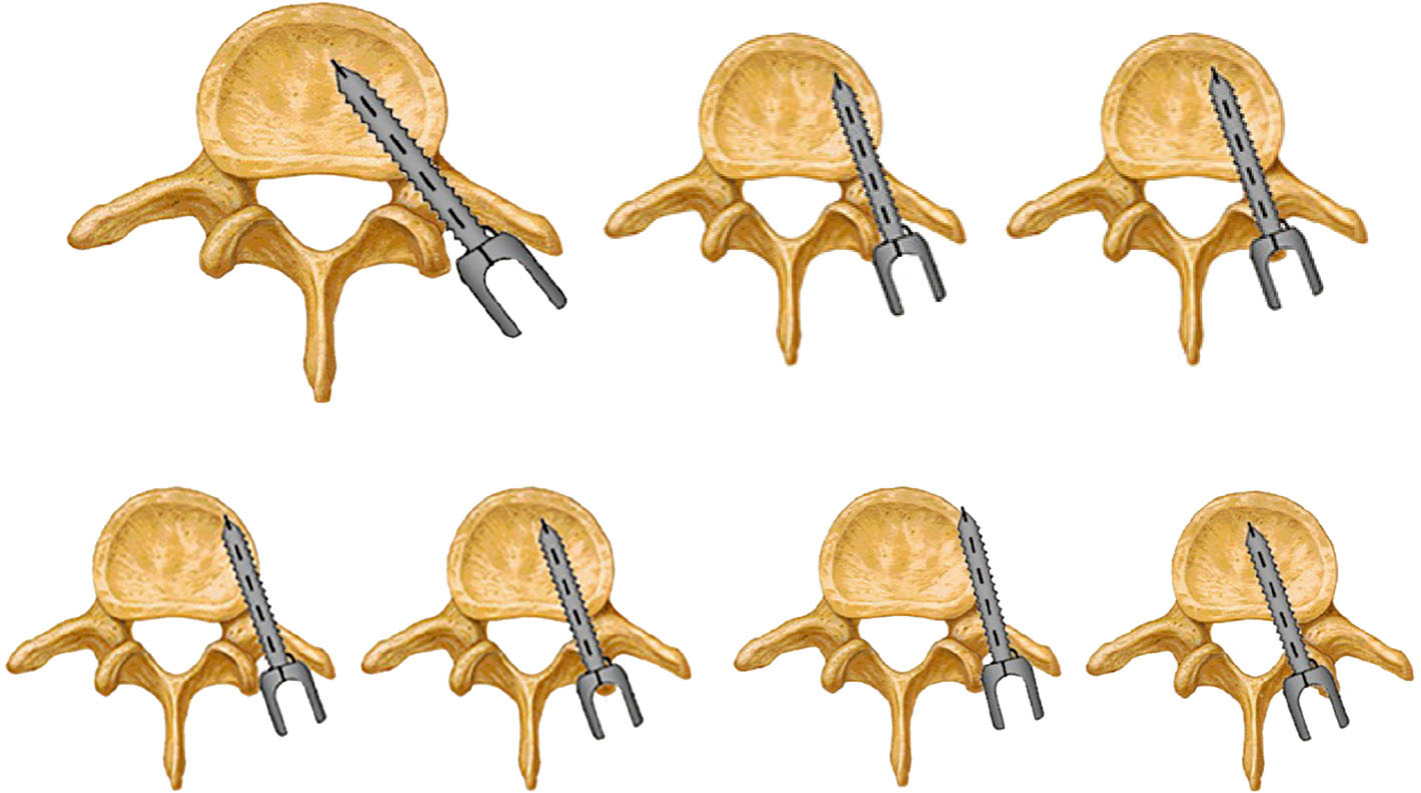
classification of pedicle screws. Grade 0: accurate pedicle screw; Grade 1: pedicle screws with < 2 mm perforation; Grade 2: pedicle screws with 2–4 mm perforation, Grade 3: pedicle screws with > 4 mm perforation

Radiographic measurements included C7–S1 sagittal vertical axis (SVA), regional kyphotic angle (RKA, Fig. 4), thoracic kyphosis (TK), and lumbar lordosis (LL). The measurement methods of RKA were shown in Fig. 4. The correction rate was calculated by [(preoperative RKA - postoperative RKA)/preoperative RKA] × 100%. All the patients in our study underwent CT scans at 3 months, and for some cases who did not acquire solid fusion, the patients accepted CT scans at 6 months follow-up. Accuracy of pedicle screws was divided into four grades [19]: G0: no violation, G1: < 2 mm perforation, G2: 2–4 mm perforation, and G3: > 4 mm perforation (Fig. 5).

### Statistical methods

SPSS 12.0 statistical software (SPSS Inc., USA) was used for statistical analysis. The t-test was used to analyze the changes of RKA, SVA, LL, TK before and after operation. A 2-sided *P* < 0.05 was considered as statistical significance.

## Results

The operation time in group A and B was 280 ± 50 min and 210 ± 30 min (P < 0.05), respectively. The estimated blood loss (EBL) in group A and B was 310 ± 70 ml and 630 ± 40 ml (P < 0.05), respectively. In group A, routine PPSs were used in 33 cases, and MIS bone cement reinforced pedicle screw system (1 case) with soft extended slice (Zina MIS Spine System, Shanghai Sanyou Medical CO., LTD) were used in one revision case in order to increase fixation strength and avoid the screws loosening. Three cases use PEEK cages at the osteotomy site. In group B, routine pedicle screws were used in all cases. No neuromonitoring abnormality was observed during the surgeries in both groups. No segmental vascular and nerve function damage, leakage of cerebral spinal fluid (CSF), and other complications were observed during and after the operatios in groups A and B.

The pre- and post-operation radiographic and clinical data were shown in Table 2. No significant difference in pre- and post-operation data was observed between the two groups. In groups A and B, RKA, SVA, and LL were all significantly improved after the operation (*P* < 0.05). The average correction rate in groups A and B was 64.5, and 66.3% respectively (*P* > 0.05).

**Table 2:** Clinical outcomes of PTK in group A and B

	RKA (^**0**^)		LL (^**0**^)		TK (^**0**^)		SVA (cm)		VAS of back pain	
	Pre-op	Post-op	Pre-op	Post-op	Pre-op	Post-op	Pre-op	Post-op	Pre-op	Post-op
**Group A**	30.7 ± 6.0	10.9 ± 1.7*	18.7 ± 11.3	26.4 ± 7.1*	22.1 ± 9.0	26.4 ± 7.1	(11.7 ± 6.5)	-(1.2 ± 3.5) *	8.6 ± 1.3	1.6 ± 1.0*
**Group B**	31.4 ± 3.1	11.6 ± 1.4*	17.9 ± 8.3	27.8 ± 9.2*	21.5 ± 6.1	18.4 ± 3.0	(13.3 ± 7.5)	-(1.6 ± 5.2) *	8.3 ± 1.2	3.0 ± 1.1*
**P**	> 0.05	> 0.05	> 0.05	> 0.05	> 0.05	> 0.05	> 0.05	> 0.05	> 0.05	< 0.05*

*p < 0.05; *RKA* regional kyphosis angle, *LL* Lumbar lordosis, *TK* Thoracic kyphosis, *SVA* sagittal vertical axis

Totally 272 PPSs were inserted in 34 cases in group A. In group B, a total of 208 pedicle screws were inserted in 26 cases. CT scan showed that 15 PPSs (5.5%) were Grade 1 and the other 257 PPS were Grade 0 screws. While there were 2 (0.9%) Grade 2 pedicle screws, 12 (5.7%) Grade 1 pedicle screws, and 194 Grade 0 pedicle screws in group B.

The average follow-up in groups A and B was 25.2 ± 7.6 months and 30.6 ± 2.7, respectively. No correction loss, internal fixation device loosening, fracture, and other complications were observed in both groups. Six months follow-up CT showed that all cases acquired solid bone fusion after surgeries. In group A and B, the patients with neurological dysfunction before surgeries were all recovered to ASIA grade E in the last follow-up. The VAS score of back pain reduced significantly from 8.6 ± 1.3 to 1.6 ± 1.0 at the last follow-up from before operation in group A (*P* < 0.05), while The VAS score of back pain significantly reduced from 8.3 ± 1.2 to 3.0 ± 1.1 at the last follow-up in group B (P < 0.05). VAS for back pain was better in group A than that in group B.

## Discussion

With the development of minimally invasive technique and instrumentation innovation, a growing number of complex deformities can be managed with hybrid approaches. Wang reported the clinical results of Mini-open pedicle subtraction osteotomy with percutaneous instrumentation as a treatment of severe adult spinal deformities [17]. Coronal alignment, lumbar lordosis, lumbar Cobb angle, pelvic tilt, and sagittal vertical axis all improved significantly, and no case of symptomatic proximal junction kyphosis. The hybrid surgery acquired similar clinical results with open surgery. However, until now there are few papers reporting the mini-open osteotomy plus PPS fixation for post-traumatic thoracolumbar kyphosis. In this series, mini-open incision Schwab grade 4 osteotomy (egg-shell technique) in a combination of PPS was used for the treatment of post-traumatic thoracolumbar kyphosis. The EBL and VAS for low back pain were much less in group A at the last follow-up, while the correction rate had no significant difference between the two groups.

The authors reported an expanded eggshell procedure, which combined with a closing-opening technique to treat thoracic and thoracolumbar angular kyphosis [10]. In this series, based on eggshell procedure, a modified Schwab grade 4 osteotomy technique was used with satisfactory clinical results. There were a few advantages to this technique in the post-traumatic thoracolumbar kyphosis series. Firstly, there is no need to remove all the anterior and lateral cortical bone of the vertebral body, which can reduce the blood loss significantly, and the remaining cortical bones would not affect the effect of deformity correction. Secondly, the affected vertebral body’s shape is like a trapezoid or triangle (lower height at the anterior vertebral body), which makes it easier to remove the upper-level intervertebral discs and cancellous bone of the vertebral body through the pedicle. Thirdly, the remaining cortical bone could be used as a “bone cage” to facilitate a large amount of bone grafting and maintain the mesh position. Fourthly, only pedicle, disc, and partial vertebral body need to be removed, thus the bone defect is usually very small after correction in Schwab grade 4 osteotomy procedure, only 3 cases need PEEK cages in this series. Stability reconstruction can be easily acquired solid bone-on-bone fusion with auto bone graft. In this series, no segmental vascular and nerve function damage, leakage of cerebral spinal fluid (CSF) and other complications were observed during and after operation. The mean blood loss was only 310 ± 70 ml. The postoperative RKA was 10.9 ± 1.70. The average correction rate was 64.5%. No correction loss, no internal fixation device loosening, fracture, and other complications were observed.

Traditional deformity correction surgeries need a long incision to expose all fixed levels. As Schwab grade 4 osteotomy procedure can acquire solid anterior bone-on-bone fusion, it is not necessary to perform posterior fusion for all fixed levels, which makes it possible to use PPSs for upper and lower fixing levels. In this series, only 5-8 cm midline incision was enough to expose the spinous process and lamina of the affected segment. Combined usage of minimally invasive pedicle screws can significantly reduce paravertebral muscle stripping, decrease intraoperative bleeding, and effectively attenuate surgical trauma. In this series, the EBL and VAS for low back pain at last follow-up were much less in group A.

In this series, all percutaneous pedicle screws were cannulated screws with soft extended slice and could be inserted percutaneously with 1.5 cm incision each. PPSs were inserted before grade 4 osteotomy, which make it possible for temporary fixation during the osteotomy procedure. Moreover, the PPSs screws had soft extended slices. During the osteotomy procedure, the soft slices can be pushed aside in order to avoid interruption of the osteotomy procedure. MIS bone cement reinforced pedicle screw system (1 case) with soft extended slice was used in one revision case in order to increase fixation strength and avoid the screws loosening.

The misplacement rate of open thoracic and lumbar pedicle screws was up to 42%, while neurological injury occurred in 2 ~ 11% patients [20, 21]. Wiesner [22] reported that misplacement rate of PPS was 6.6%, only 1 PPS penetrated medial pedicle cortex and caused minor neurological deficit. Oh et al. [23] compared the accuracy of 558 open pedicle screws (OPS) with 498 PPS using CT. The accuracy rate of OPS (13.4%) and PPS (14.3%) had no statistical difference. In the OPS group, the incidence of lateral penetration was relatively high (66.7% vs. 43.7%), while in the PPS group, the incidence of superior, medial, and inferior penetrations was relatively high. Unlike scoliosis cases, the pedicels anatomy of post-traumatic thoracolumbar kyphosis patients was usually normal, the PPS insertion under fluoroscopy was not a big technique challenge. In this study, the security of PPSs was also evaluated by CT. Among all PPSs, only15 Grade 1 screws (5.5%) were identified. Meanwhile, post-operative CT showed that the misplace rate was 6.6% (two grade 2 and twelve grade 1 pedicle screws) in group B. The results showed that no significant difference was observed between groups A and B in misplacement rate. Percutaneous pedicle screws were safe and accurate for kyphosis deformity cases.

Our results show that Mini-open SRS-Schwab grade 4 osteotomy combined with percutaneous pedicle screws fixation is a minimally invasive, safe, and effective method for PTK treatment. However, more cases and long-term follow-up result are needed to confirm the efficacy of this technique.

## Conclusion

Schwab grade 4 osteotomy combined with percutaneous pedicle screws fixation is a minimally invasive, safe and effective method for PTK treatment.

## Data Availability

The datasets used and/or analyzed during the current study are available from the corresponding author on reasonable request.

## Abbreviations

PPS: Percutaneous pedicle screws
PTK: Post-traumatic thoracolumbar kyphosis
RKA: Regional kyphosis angle
EBL: Estimated blood loss
CSF: Cerebral spinal fluid
SVA: Sagittal vertical axis
LL: Lumbar lordosis
VAS: Visual Analogue Scale
SPO: Smith-Petersen osteotomy
PSO: Pedicle subtraction osteotomy
COWO: Closing-opening wedge osteotomy
VCR: Vertebral column resection
MIS: Minimally invasive surgery
ASD: Adult spinal deformity
ASIA: American Spinal Injury Association
IOM: Inter-operative Monitoring
SEPs: Somatosensory evoked potentials
MEPs: Motor evoked potentials
TK: Thoracic kyphosis
